# Translatable tool to quantitatively assess the quality of red blood cell units and tailored cultured red blood cells for transfusion

**DOI:** 10.1073/pnas.2318762121

**Published:** 2024-03-04

**Authors:** Lars Kaestner, Peter Schlenke, Marieke von Lindern, Wassim El Nemer

**Affiliations:** ^a^Theoretical Medicine and Biosciences, Saarland University, Campus Saarland University Hospital, Homburg/Saar 66424, Germany; ^b^Dynamics of Fluids, Experimental Physics, Saarland University, Saarbruecken 66123, Germany; ^c^Department of Blood Group Serology and Transfusion Medicine, Medical University of Graz, Graz 8036, Austria; ^d^Landsteiner Laboratory, Amsterdam University Medical Center, University of Amsterdam, Amsterdam 1105AZ, The Netherlands; ^e^Department Hematopoiesis, Sanquin Blood Supply Foundation, Amsterdam 1066CX, The Netherlands; ^f^Etablissement Français du Sang Prevence Alpes Côte d’Azur-Corse, Aix Marseille University, Centre national de la recherche scientifique (CNRS), Anthropologie bio-culturelle, Droit, Ethique et Santé (UMR 7268), Globule Rouge laboratory of excellence (GR-Ex), Marseille 13005, France

**Fig. 1.**
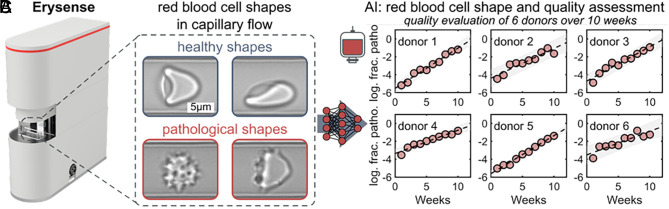
Erysense device, principle of measurement, and AI–based analysis of single-cell microfluidic flow behavior of stored RBCs. (*A*) Image of the Erysense device. A micro-structured chip without the need to connect any tubes (i.e., no cleaning requirements, no risk for contamination) requires very small volumes of packed RBCs (1 µL). It is an all-integrated “point-of-care” device with a footprint of an Espresso machine. (*B*) Representative images of healthy croissant-shaped (*Top Left*) and slipper-shaped RBCs (*Top Right*) at low (1 mm/s) and high (10 mm/s) velocities, respectively. Representative images of pathological RBC shapes during capillary flow (Lower images). Flow is from *Left* to *Right* in all images. (*C*) AI-based evaluation of donor-dependent (donors 1 to 6) changes in the RBC microcapillary flow behavior. Dashed black lines correspond to linear fits. Gray areas indicate an estimate of a 95% prediction interval. The *y*-axis shows the logarithm of the fraction of pathological RBC shapes based on the shape phase diagrams. Panels *A* and *B* are reproduced with permission from Recktenwald et al. 2022 (4) and panel *C* with permission from Lopes et al. 2023 (6).

Recently, Isiksacan et al. introduced in their perspective article the assessment of stored red blood cells (RBCs) through lab-on-a-chip (LOC) technologies for precision transfusion medicine (1). The article presents a timely vision and we share and support the central message; however, a broader perspective is warranted in light of the European experience. Europe accounts for more than 35% of the annual 56 million blood transfusions worldwide registered by the World Health Organization (2). It is thus worthy to emphasize that the European Commission will impose quality control requirements for stored RBC through the regulation of quality and safety standards for substances of human origin (SoHO) intended for human application (3) repealing previous regulations (Directive 2002/98EC, 2004/23EC). The proposal of Isiksacan et al. (1) is thus not simply visionary but a compulsory requirement and calls for the timely implementation of novel technologies. One such LOC device is already commercially available on the European market specifically designed for RBC quality assessment (Erysense, Cysmic GmbH, Germany) (4) and meets most of the requirements defined by Isiksacan et al. (1). In contrast to other more academic approaches, chip geometry is kept simple by design (4): Capillary flow is mimicked with no constrictions and thus avoids the risk of channel clogging (Fig. 1*A*). Furthermore, several relevant cellular properties govern RBC morphology in flow, including cytosolic viscosity (and thus hydration status), cytoskeleton integrity, and also plasma membrane properties. This means shape changes during flow (Fig. 1*B*) reflect the biophysical and biochemical status of RBC and thus reveal signs of storage lesions. It was shown that the use of Erysense in combination with AI–based prediction models (5) can monitor RBC quality during standardized storage conditions, revealing i) a constant decline in RBC quality and ii) a donor-related variability (6) (Fig. 1*C*). If incorporated into routine blood banking, regular testing of blood units may not only impact clinical outcomes from a personalized medicine perspective (1) but would also reduce the number of blood units discarded owing to the current use of one-size-fits-all expiration dates [approximately 1.4 million units annually worldwide (2)]. To fully realize the potential of such LOC devices, we urge that the use of AI in “In vitro diagnostic” devices needs simplifying; current approval procedures by regulatory bodies (e.g., Food and Drug Administration; Medical Drug Administration) do not yet facilitate the advantages of the “self-improving” properties of AI (6, 7).

Last but not least, an additional aspect should be considered as part of the vision of precision transfusion medicine extending the perspective of Isiksacan et al. (1): A number of European research institutions and companies focus on the in vitro production of RBCs at a scale relevant for transfusions (8–10). These cultured RBCs can be “designed” in terms of optimizing allosteric properties. Table 1 summarizes the conceptual outline and provides the state-of-the-art. In future, the transfusion of tailored in vitro-produced RBCs may represent the most sophisticated approach for personalized transfusions.

**Table 1.** Conceptual use of in vitro produced RBCs in personalized transfusion medicine

Order	Requirement/procedure	Example	State-of-the-art
1	Patient (group)-specific allosteric requirement(s)	Rare blood group types and allo-immunized patients	Undertreated patients due to lack of matching blood; need for cultured RBCs
2	Source of precursor cells	Inducible pluripotent stem cells or embryonic stem cells	Isolation protocols are well established; use of embryonic stem cells ethically banned in some countries
3	Genetic manipulation	Knock-out or replacement of particular proteins	CRISPR/Cas systems allow for straightforward manipulations
4	Expansion and differentiation	Upscaling in bioreactors, culture medium composition, postprocess purification of enucleated cells	Stirred bioreactors and perfusion, free of animal/human components, leukocyte depletion filters
5	Quality control	Investigation of capillary flow properties	Under development; clinical trials with the transfusion of cultured RBCs are ongoing
6	RBC conservation	Storage conditions, freezing protocols	Available protocols for erythrocytes may not suit cultured reticulocytes
7	Quality control	Capillary flow properties	So far only based on hemolysis, but potential for LOC devices like Erysense (Fig. 1)
8	Personalized transfusion upon request	For planned surgeries McLeod patients can get autologous blood transfusions	Although a “misuse “, there are established protocols for autologous blood doping based on frozen blood samples (can still be refined)
